# PADI4 and the HLA-DRB1 shared epitope in juvenile idiopathic arthritis

**DOI:** 10.1371/journal.pone.0171961

**Published:** 2017-02-09

**Authors:** Kaori Hisa, Masakatsu D. Yanagimachi, Takuya Naruto, Takako Miyamae, Masako Kikuchi, Rhoki Hara, Tomoyuki Imagawa, Shumpei Yokota, Masaaki Mori

**Affiliations:** 1 Department of pediatrics, Yokohama City Graduate School of Medicine, Yokohama, Japan; 2 Department of infectious disease and immunology, Kanagawa Children’s Medical Center, Yokohama, Japan; 3 Department of Pediatrics, Tokyo Medical and Dental University, Tokyo, Japan; 4 Department of Lifetime Clinical Immunology, Graduate School of Medical and Dental Sciences, Tokyo Medical and Dental University, Tokyo, Japan; JAPAN

## Abstract

**Objective:**

Both genetic and environmental factors are associated with susceptibility to juvenile idiopathic arthritis (JIA). Many studies have reported that both a ‘shared epitope’ (SE) encoded by several HLA-DRB1 alleles and the peptidyl arginine deiminase type 4 (PADI4) gene polymorphisms are associated with susceptibility to rheumatoid arthritis (RA). However, it is uncertain whether JIA and RA share the latter genetic risk factor. Therefore, here we investigated relationships between HLA-SE and PADI4 polymorphisms with clinical subtypes of JIA.

**Methods:**

JIA patients (39 oligoarthritis, 48 RF-positive polyarthritis, 19 RF-negative polyarthritis and 82 systemic) and 188 healthy controls were genotyped for HLA-DRB1 by PCR-sequence-specific oligonucleotide probe methodology. Three PADI4 gene single nucleotide polymorphisms (SNPs), rs2240340, rs2240337 and rs1748033, were genotyped using TaqMan SNP Genotyping Assays.

**Results:**

Frequencies of the HLA-SE were higher in RF-positive polyarticular JIA than in healthy controls. RF-positive polyarticular JIA was associated with HLA-SE (OR = 5.3, 95% CI = 2.5–11.9, pc < 0.001). No associations were found between clinical subtypes of JIA and PADI4 allele frequency. Nonetheless, rs2240337 in the PADI4 gene was significantly associated with anti-cyclic citrullinated peptide antibody (ACPA)-positivity in JIA. The A allele at rs2240337 was a significant risk factor for ACPA positivity in JIA (OR = 5.6, 95% CI = 1.71–23.7 pc = 0.03).

**Conclusion:**

PADI4 gene polymorphism is associated with ACPA-positivity in JIA. The association of HLA-SE with RF-positive polyarticular JIA as well as RA is confirmed in Japanese. Thus, HLA-SE and PADI4 status both influence JIA clinical manifestations.

## Introduction

Juvenile idiopathic arthritis (JIA) is defined as a chronic arthritis developing in children <16 years of age and persisting for ≥6 weeks. According to the International League of Associations for Rheumatology (ILAR) classification criteria for JIA, it has 7 subtypes [1]. The 4 major subtypes are oligoarthritis, rheumatoid factor (RF)-positive polyarthritis, RF-negative polyarthritis and systemic arthritis. The major pathology of oligoarthritis and polyarthritis is articular inflammation and joint destruction. RF-positive polyarthritis is considered to be a counterpart of adult rheumatoid arthritis (RA) [2]. In contrast to the above forms of JIA, the major pathology of systemic JIA is systemic inflammation, which is considered similar to adult Still’s disease [3,4].

In RA and JIA, both genetic and environmental factors are associated with disease susceptibility [5]. HLA class II gene polymorphisms are considered the most influential for RA susceptibility [6]. Many studies have reported the association of a ‘shared epitope’ (SE) encoded by several HLA-DRB1 alleles with RA susceptibility in adults [7]. Similarly, an association between HLA-SE and susceptibility to JIA has been reported in Caucasians [8]. We have previously reported that HLA-DRB1*04:05, a major SE-containing allele, is associated with polyarticular JIA also in the Japanese population [9].

More recently, a number of RA susceptibility genes outside of the HLA region have been identified by genome-wide association studies (GWAS) [10,11]. One of these, peptidyl arginine deiminase type 4 (PADI4) was first reported in Japanese RA patients [12,13], and subsequently confirmed in several Asian groups and subgroups of Europeans [14–17]. PADI4 is one member of PADI gene family. It codes for enzymes responsible for the posttranslational conversion of arginine residues into citrulline. It was indicated that an RA susceptibility haplotype in PADI4 was associated with increased stability of PADI4 mRNA [13]. And it could lead to accumulation of PADI4 protein, with subsequent increases in citrullinated proteins and enhanced production of autoantibodies against these citrullinated peptides [18].

PADI4 mRNA is detected in hematological cells and pathological synovial tissues [19,20]. And it was reported that PADI4 significantly overexpressed in the blood cells of RA patients [21]. Moreover, PADI4 have a nuclear localization signal, which affects the expression control of various genes [22]. PADI4 may have various role in the immune system and associated with development of autoimmune disease.

In each of the JIA subtypes, age of onset, clinical course and serological findings are different, which may be accounted for by different influences of the genetic background. However, it is uncertain whether JIA (particularly the RF-positive polyarthritic form) and RA share any genetic risk factors other than HLA-SE. There are no reports that PADI4 risk alleles are involved in JIA disease susceptibility. In the present study, which includes our previous cohort [9], we investigated relationships between HLA-SE and PADI4 polymorphisms, and clinical subtypes of JIA in the Japanese population.

## Materials and methods

### Study population

Patients were eligible if they met the ILAR classification criteria for JIA. A total of 188 JIA patients (39 oligoarthritis, 48 RF-positive polyarthritis, 19 RF-negative polyarthritis and 82 systemic), comprising 59 boys and 129 girls, was enrolled in this study and followed at the Yokohama City University Hospital between December 2006 and December 2009. This cohort included the 106 oligo- and poly-articular JIA patients who were described in our previous study [9]. Clinical data including age at onset, gender, RF and anti-cyclic citrullinated peptide antibody (ACPA) status were reviewed.

We conducted this study in accordance with the Declaration of Helsinki and with the approval of the Ethics Committee of the Yokohama City University School of Medicine. Written informed consent was obtained from each patient and/or their guardian. (Approval number: A090528002)

### HLA genotyping

Genomic DNA was isolated from peripheral blood using the QIAamp DNA Mini kit (Qiagen K.K., Tokyo, Japan). JIA patients and healthy adult controls were genotyped for HLA-DRB1 using PCR sequence-specific oligonucleotide probes (SSOP) by the Luminex method with Genosearch HLA-A, -B and -DRB1 Ver. 2 (Medical & Biological Laboratories Co., Ltd. Nagoya, Japan), as described previously [9]. HLA-DRB1*01:01, *04:01, *04:04, *04:05, *04:10, *10:01, *14:02 and *14:06 were regarded as HLA-SE alleles [23].

### PADI4 genotyping

Three single nucleotide polymorphisms (SNPs), rs2240340, rs2240337 and rs1748033 in the PADI4 gene were selected based on previous research [12,13]. Genotyping for these in 188 JIA patients and 188 healthy adult controls was performed using TaqMan SNP Genotyping Assays (AB assay ID: C__16176717_10 for rs2240340, C___3123009_1 for rs2240337 and C___7541083_1 for rs1748033). These SNPs were analyzed by real-time PCR using the AB7500 Real Time PCR system (Applied Biosystems, Foster City, CA, USA) under the conditions recommended by the manufacturer. Allele discrimination was accomplished using SDS software version 1.4 (Applied Biosystems).

### Statistical analysis

The statistical significance of the differences in the frequencies of HLA-DRB1 alleles or PADI4 gene polymorphisms between JIA subtypes was evaluated by Fishers exact test. A corrected P-value (Pc) was calculated by multiplying the P-value by the number of HLA-DRB1 alleles tested at each locus. For the PADI4 gene polymorphisms, we examined 3 SNPs and used a total of 5 independent tests.

## Results

### Patients’ characteristics

Characteristics of the patients studied are shown in Table 1. Patients comprised 39 children with oligoarthritis, 48 with RF-positive polyarthritis, 19 with RF-negative polyarthritis and 82 with systemic arthritis. The mean age at onset of oligoarthritis was 5.6 years, RF-positive polyarthritis was 8.2 years, RF-negative polyarthritis was 7.1 years and systemic arthritis 5.0 years.

**Table 1:** Clinical characteristics of JIA patients.

	Oligo articular JIA (n = 39)	RF positive, polyarticular JIA (n = 48)	RF negative, polyarticular JIA (n = 19)	Systemic JIA (n = 82)
Age at JIA onset (years,mean)	5.6	8.2	7.1	5
Gender (female,%)	35 (90%)	40(83%)	10(53%)	44 (53%)
ANA (>1:160,%)	16 (41%)	19(40%)	3(16%)	3/78 (4%)
RF (>14.0 (IU ml-1),%)	9 (23%)	48(100%)	0(0%)	-
Anti-CCP (>4.5(U ml-1),%)	8 (21%)	40(83%)	0(0%)	0/43 (0%)

### HLA-DRB1 and JIA subtypes

188 healthy controls was genotyped for HLA-DRB1 to determine associations of HLA-DRB1 and HLA-SE with JIA subtype susceptibility. According to ILAR classification criteria for JIA, RF-positive oligoarticular JIA is classified as “undifferentiated”. Thus, such cases were excluded from the oligoarthritis group in HLA association studies. RF-positive polyarticular JIA was significantly associated with HLA-DRB1*04:05 and HLA-SE (OR = 5.1, 95% CI = 2.5–11, pc < 0.001; OR = 5.3, 95% CI = 2.5–11, Pc < 0.001, respectively) (Table 2). In contrast, frequencies of HLA-DRB1*04:05 and HLA-SE were not higher in the other types of JIA patients.

**Table 2:** Association of HLA-DRB1*04:05 and HLA-SE with susceptibility to JIA subtypes.

HLA-DRB1*0405	Genotype (*0405/any)	OR	95% CI	P-value	Pc
control (n = 188)	40 (21.3%)	-	-	-	-
Oligoarticular JIA (n = 30)	1(3.3%)	0.1	0.01–0.82	0.02	NS
RF positive, polyarticular JIA (n = 48)	28 (58.3%)	5.1	2.50–10.7	<0.001	<0.001
RF negative,polyarticular(n = 19)	4(21.1%)	1	0.30–4.42	0.98	NS
RF negative(oligo+poly)(n = 49)	5(10.2%)	0.4	0.86–8.17	0.078	NS
Systemic JIA (n = 82)	21 (25.6%)	1.3	0.66–2.42	0.43	NS
HLA-SE	Genotype (SE/any)	OR	95% CI	P-value	Pc
control (n = 188)	68 (36.2%)	-	-	-	-
Oligoarticular JIA (n = 30)	6(20.0%)	0.4	0.14–1.18	0.082	NS
RF positive polyarticular JIA (n = 48)	36 (75.0%)	5.3	2.47–11.9	<0.001	<0.001
RF negative,polyarticular(n = 19)	5(26.3%)	0.6	0.17–1.96	0.39	NS
RF negative(oligo+poly)(n = 49)	15(30.6%)	0.8	0.37–1.60	0.47	NS
Systemic JIA (n = 82)	33 (40.2%)	1.8	0.67–2.09	0.59	NS

**S**E, shared epitope: HLA-DRB1*04:05,01:01,04:01,04:10,10:01,14:02,14:06

### PADI4 polymorphisms and JIA subtypes

Frequencies of PADI4 gene polymorphisms studied in JIA patients and controls are shown in Table 3. There were no associations between clinical subtypes of JIA and PADI4 gene polymorphisms. Nonetheless, the PADI4 SNPs were significantly associated with ACPA positivity in JIA (Table 4). Because the ACPA status of all systemic JIA patients measured in this study was negative (0/43), systemic JIA was excluded from the data in Table 4. Hence, the A allele at rs2240337 is a significant risk factor for ACPA positivity in oligo- and poly-articular JIA (OR = 5.6, 95% CI = 1.7–24 Pc = 0.03). Finally, there were no associations between HLA-SE and PADI4 gene polymorphisms in oligo- and poly-articular JIA (Table 5).

**Table 3:** Association between PADI4 gene polymorphisms and susceptibility to JIA subtypes.

rs2240340	G allele	A allele	MAF	OR	95% CI	P	Pc
Control (n = 188)	223	153	0.41	-	-	-	-
Oligoarticular JIA (n = 30)	37	23	0.38	0.9	0.49–1.64	0.73	NS
RF positive, polyarticular JIA (n = 48)	49	47	0.49	1.4	0.87–2.25	0.17	NS
RF negative,polyarticular(n = 19)	24	14	0.37	0.9	0.39–1.78	0.64	NS
RF negative,oligo+poly articular(n = 49)	61	37	0.38	0.9	0.54–1.43	0.6	NS
Systemic JIA (n = 82)	92	72	0.44	1.1	0.77–1.68	0.51	NS
rs2240337	G allele	A allele	MAF	OR	95% CI	P	Pc
Control (n = 188)	350	26	0.07	-	-	-	-
Oligoarticular JIA (n = 30)	57	3	0.05	0.7	0.13–2.43	0.45	NS
RF positive, polyarticular JIA (n = 48)	85	11	0.12	1.7	0.75–3.82	0.14	NS
RF negative,polyarticular(n = 19)	37	1	0.03	0.4	0.01–2.36	0.25	NS
RF negative,oligo+poly articular(n = 49)	94	4	0.04	0.6	0.14–1.71	0.21	NS
Systemic JIA (n = 82)	149	15	0.18	1.4	0.65–2.74	0.38	NS
rs1748033	G allele	A allele	MAF	OR	95% CI	P	Pc
Control (n = 188)	239	137	0.36	-	-	-	-
Oligoarticular JIA (n = 30)	42	18	0.30	0,7	0.39–1.39	0.33	NS
RF positive, polyarticular JIA (n = 48)	55	41	0.43	1.3	0.80–2.10	0.29	NS
RF negative,polyarticular(n = 19)	26	12	0.32	0.8	0.36–1.72	0.55	NS
RF negative,oligo+poly articular(n = 49)	68	30	0.31	0.8	0.46–1.27	0.28	NS
Systemic JIA (n = 82)	120	44	0.27	0.6	0.42–0.97	0.03	NS

**Table 4:** Association between PADI4 gene polymorphisms and ACPA positivity in oligo- and poly- articular JIA patients (n = 106).

		Anti-CCP(-) (<4.5U ml-1) (n = 58)	Anti-CCP (+) (>4.5U ml-1) (n = 48)	OR	95% CI	P	Pc
rs2240340	allele	75	46	2	1.1–3.6	0.018	NS
	recessive	26	11	2.7	1.1–7.1	0.024	NS
	dominant	49	35	2	0.70–6.0	0.158	NS
rs2240337	allele	112	80	5.6	1.7–24	0.002	0.03
	recessive	54	32	6.6	1.9–30	<0.001	<0.001
	dominant	-	-	-	-	-	-
rs1748033	allele	80	52	1.9	1.0–3.4	0.03	NS
	recessive	30	14	2.6	1.1–6.4	0.029	NS
	dominant	53	38	2.8	0.78–11	0.095	NS

Recessive: GG versus (GA/AA), dominant: (GG/GA) versus AA

**Table 5:** Association between PADI4 gene polymorphisms and SE positivity in oligo- and poly- articular JIA (n = 106).

		GG	GA/AA	OR	95% CI	P-value
rs2240340	SE-	20	31	1.4	0.60–3.5	0.42
	SE+	17	38	-	-	-
rs2240337	SE-	42	9	1.2	0.39–3.5	0.81
	SE+	44	11	-	-	-
rs1748033	SE-	23	28	1.3	0.57–3.1	0.56
	SE+	21	34	-	-	-

## Discussion

Susceptibility to RA is influenced by both genetic and environmental factors such as smoking. Many studies have determined that the major RA disease susceptibility genes are the HLA class II alleles. The shared epitope (SE) hypothesis for risk of RA is well-established [7], indicating that multiple HLA-DRB1 alleles are the strongest known genetic risk factors for RA by virtue of encoding a shared amino acid sequence, known as a shared epitope, SE [6]. Several studies have also reported associations between the genetic background and JIA susceptibility [5], including associations with HLA alleles [24–29]. An association between HLA-SE and susceptibility to JIA has been confirmed in 204 RF- or ACPA-positive Caucasian JIA patients [8].

The contribution of HLA to RA susceptibility, however, accounts for only about 30% of incidence, implying that genes other than those in the HLA region are involved; some estimates suggest as many as 100. Other genes influencing RA susceptibility have now been identified, such as PADI4, PTPN22 and CTLA4. Numerous non-HLA JIA susceptibility genes have also been imputed using GWAS [11]. Variants at the PTPN22, STAT4, TNF-α, TNFAIP3, MIF, WISP3, SLC11A1 and IL2-Ra loci have been reported as risk factors for JIA by several investigators [5], although it was also reported that several of these are not necessarily shared between different ethnic groups [10,30]. Thus, there are likely to be different genetic risk factors for JIA in different ethnic groups. Therefore, here we sought an influence of HLA-SE and PADI4 on JIA susceptibility in Japanese, because both HLA-SE and PADI4 were reported as significant genetic risk factors for RA independent of ethnicity [14,15,31].

We previously reported an association of HLA-A*02:06 with JIA accompanied by uveitis and of HLA-DRB1*04:05 with polyarticular JIA [9]. In the present study, we confirmed the association between HLA-SE and RF-positive polyarticular JIA in Japanese. However, we found that HLA-SE was not associated with oligoarticular or systemic JIA in our cohort. Recently, it was reported that five amino acids in three HLA molecules, including three amino acid positions (11, 71 and 74) in HLA-DRB1, were associated with RF-seropositive RA by the HLA-imputation method [32]. It should therefore be evaluated whether these HLA amino acids are also associated with JIA susceptibility in future.

In addition to RF, ACPA is the most specific serologic marker in adult RA with a specificity of 95% and a sensitivity of 80%, similar to RF [33,34]. Considering all JIA subtypes together, ACPA was detected in 1.8–28.6% of patients, a low frequency compared to RA. However, ACPA was present in 70–90% of RF-positive polyarticular JIA patients [35]. Bone destruction is more severe in these ACPA-positive patients [36]. These results suggest that ACPA-positive polyarticular JIA may be similar to RA with regard to pathogenetic processes.

PADI4, a member of the PADI family, was first reported to be associated with RA in a Japanese population [12,13]. It encodes a peptidyl arginine deiminase responsible for the posttranslational conversion of arginine residues into citrulline. We investigated associations between PADI4 gene polymorphisms and ACPA positivity in JIA in our Japanese population. The stability of PADI4 mRNA differs according to these gene polymorphisms, which may represent the mechanism by which it influences the production of ACPA [13]. To the best of our knowledge, there are no reports that PADI4 risk alleles are involved in JIA disease susceptibility. It is likely that PADI4 is also a JIA susceptibility gene in ethnic groups other than Japanese, especially in ACPA-positive JIA. This hypothesis needs further exploration.

We found no association between HLA-SE and PADI4 in JIA patients, implying that HLA-SE and PADI4 are independent JIA susceptibility genes. However, an association between HLA-SE and citrullination in the pathogenesis of RA has been noted [37]. The electropositive P4 pocket of HLA-DRB1*04:01/04 can accommodate citrulline-containing epitopes, and the CD4^+^ T cell repertoire for citrullinated antigens is increased in RA patients harboring HLA-DRB1*04:01/04. These potential pathogenetic mechanisms may also contribute to JIA. Further study is needed to determine whether this is the case.

In conclusion, we found an association of PADI4 gene polymorphisms with ACPA-positivity in JIA, as was already known for RA. We also confirmed the influence of HLA-SE on RF-positive polyarticular JIA in the Japanese population. Thus, JIA may be classified into clinical and genetic background-based subtypes using HLA-SE and PADI4 genotyping.
